# Does Low-intensity pulsed ultrasound treatment repair articular cartilage injury? A rabbit model study

**DOI:** 10.1186/1471-2474-15-36

**Published:** 2014-02-10

**Authors:** Shan-Wei Yang, Chien-Lin Kuo, Shwu Jen Chang, Po-Chou Chen, Yen Ting Lin, Ioannis Manousakas, Shyh Ming Kuo

**Affiliations:** 1 Department of Orthopedics, Kaohsiung Veterans General Hospital, Kaohsiung 81346, Taiwan; 2 Department of Physical Therapy, Tzu-Hui Institute Technology, Pingtung County 926, Taiwan; 3 Department of Orthopedics, Gang-Shang Armed Forces Hospital, Kaohsiung 820, Taiwan; 4 Department of Biomedical Engineering, I-Shou University, No.1, Sec. 1, Syuecheng Rd., Kaohsiung City 84001, Dashu District, Taiwan

**Keywords:** Low-intensity pulsed ultrasound, Cartilage, Injury

## Abstract

**Background:**

Low-intensity pulsed ultrasound (LIPUS) regiment has been used to treat fractures with non-union and to promote bone union in general. The effect of LIPUS on articular cartilage metabolism has been characterized. Yet, the effect of LIPUS to repair articular cartilage injury remains unclear in vivo.

**Methods:**

We designed a study to investigate the effect of LIPUS on articular cartilage repairing in a rabbit severe cartilage injury model. Eighteen rabbits were divided into three groups: Sham-operated group, operated group without-LIPUS-treatment, operated group with-LIPUS-treatment (a daily 20-minute treatment for 3 months). Full-thickness cartilage defects were surgically created on the right side distal femoral condyle without intending to penetrate into the subchondral bone, which mimicked severe chondral injury. MR images for experimental joints, morphology grading scale, and histopathological Mankin score were evaluated.

**Results:**

The preliminary results showed that the operated groups with-LIPUS-treatment and without-LIPUS-treatment had significantly higher Mankin score and morphological grading scale compared with the sham-operated group. However, there was no significant difference between the with-LIPUS-treatment and without-LIPUS-treatment groups. Cartilage defects filled with proliferative tissue were observed in the with-LIPUS-treatment group grossly and under MR images, however which presented less up-take under Alcian blue stain. Furthermore, no new deposition of type II collagen or proliferation of chondrocyte was observed over the cartilage defect after LIPUS treatment.

**Conclusion:**

LIPUS has no significant therapeutic potential in treating severe articular cartilage injury in our animal study.

## Background

Chondral injuries are common lesions of the knee joint. Variety of causes results to chondral injury, such as trauma, aging degeneration and infection. Currently, many treatments are available for chondral injury, such as physical therapy, lifestyle modification, pharmacological medications with non-steroid anti-inflammatory drugs (NSAID) or glucosamine, and intra-articular injection of Hyaluronan [1]. The very limited capability for self repair and subsequent degeneration of injuried cartilage and other articular tissues often lead to osteoarthritis, which may eventually lead to the need for total knee arthroplasty [2]. However, the better treatments for chondral injury should not only target the symptoms of the patient but also promote biological repair of the destructed articular cartilage tissue.

Ultrasound is a form of mechanical energy that can be transmitted into the biological tissue as high frequency acoustical pressure waves. It was used as a diagnostic and therapeutic tool. The therapeutic ultrasound achieves its biological result such as muscle pain relief and decrease of joint stiffness by increasing the temperature of the tissue, with intensities ranging from 1 to 3 W/cm^2^. In contrast, the intensities of diagnostic images are of much lower level (0.5 to 50 mW/cm^2^) without thermogenic and destructive actions. Low-intensity pulsed ultrasound (LIPUS) is a recommended therapy to treat fractures with non-union and promotes bone union clinically [3-8]. Application of high-intensity continuous ultrasound generates considerable heat in living tissues, whereas LIPUS (< 100 mW/cm^2^) has much lower intensity with non-thermogenic and non-destructive actions.

Previous studies presented the LIPUS enhances the endochondral ossification in the healing process of fractured bone and promote bone formation, possibly by inducing chondrocyte proliferation [4-8]. It also regulated vascular endothelial growth factor (VEGF) expression in early fracture healing phase and subsequent chondrogenesis [9]. Furthermore, some in vitro studies demonstrated the LIPUS may potentially protect cartilage by inhibiting matrix metalloproteinase-13 (MMP-13) mRNA expression, and stimulate chondrocyte proliferation and matrix production in chondrocytes [10-12]. It also has been reported to promote the mRNA expression of type II collagen, type X collagen, aggrecan, and transforming growth factor (TGF)-ß in chondrocytes [13]. In this context, the effect of LIPUS on articular cartilage metabolism has been characterized.

Most animal studies that analyze the histological and biochemical changes in osteoarthritis are anterior cruciate ligament (ACL) transection model in canines or partial meniscus resection model in rabbits or rats, which result to joint instability and induced cartilage degeneration gradually [14-18]. However, the studies had difficulties in controlling the consistency of the cartilage injuries among the animals.

We designed an experimental rabbit model of severe articular cartilage injury to evaluate the effect of cartilage repair. Surgically created defects of full-thickness cartilage were performed to build consistent severe cartilage injuries. The destruction of the full-thickness cartilage could be controlled, and the effect of cartilage repair could be evaluated quantitatively. Following the model, we investigated the effect of LIPUS on cartilage repair.

## Methods

### Materials of animal model and grouping

Eighteen female Japanese white rabbits with post-natal 12 weeks and body weight between 2.0 to 2.5 kilograms were selected into this trial. Two rabbits per cage were housed under a specific pathogen-free condition (controlled temperature of 24 ± 3°C and humidity of 55 ± 15%) and fed the same standard laboratory food ad libitum. All rabbits were allowed to move freely in the cages. Eighteen rabbits were randomly divided into three groups: 1. Sham-operated control group (sham: n = 6); 2. Operated experimental group without LIPUS treatment (without-LIPUS-treatment: n = 6); 3. Operated experimental group with LIPUS treatment (with-LIPUS-treatment: n = 6). The present investigation conforms to the Guide for the Animal Use Protocol of Institutional Animal Care and Use Committee of I-Shou University (IACUC-ISU99010).

### Surgical preparation for cartilage defect

**Figure 1:**
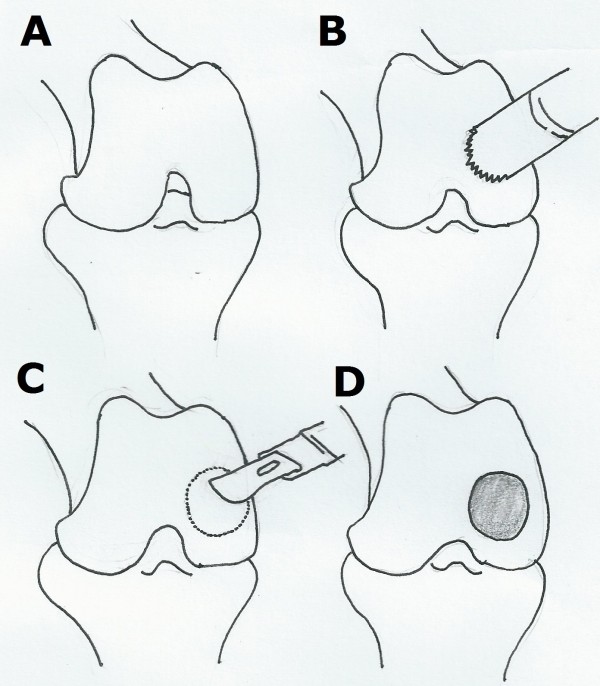
**Surgical procedures for cartilage defect. (A)** The joint was exposed after anesthesia. **(B)** trephine (5 mm in diameter) was used to create a cartilage defect with a round shape on the femoral medial condyle. **(C)** Cartilage within the circle was removed with a knife gently. **(D)** The subchondral bone was exposed as a severe chondral injury.

Cartilage defect were created on the right side distal femoral condyle of the rabbits surgically in the operated experimental group with/without LIPUS treatment. Each rabbit was anesthetized by intra-muscular injection anesthesia with Zoletil 50 (2.0 ml/kg). After the right knee joints of each rabbit were shaved and disinfected, the knee joints were exposed through a medial para-patellar approach. The patella was dislocated laterally and the knee placed in flexion 70 degrees to expose the distal femoral condyle. A trephine (5 mm in diameter) was used to create a full-thickness cartilage defect with a round shape on the femoral medial condyle without intending to penetrate into the subchondral bone. Cartilage within the circle was removed with a knife gently until the subchondral bone was exposed (Figure 1). After the surgery, the joint surface was washed with sterile saline, and both capsule and skin were sutured used Vicryl 4–0 absorbable suture and mono-filament 4–0 Nylon threads. In the sham-operated control group, the right knee joints of rabbits were exposed and incisions were closed after subluxation of the patella and washing the joint surface with saline without destruction of the cartilage. All rabbits could move freely in the cages and move outside the cage for 30 minutes every day.

### LIPUS treatment

The Exogen low-intensity pulsed ultrasound device (Smith & Nephew Inc, Memphis, TN, USA) was used on the operated right knees of each rabbit in the with-LIPUS-treatment group for a daily 20-minute treatment for 3 months after the surgery. The LIPUS device produced a 200 μs burst of 1.5 MHz acoustic sine waves with a pulse repetition frequency of 1 kHz, and provided a peak intensity of 30 mW/cm^2.^ The knees of each rabbit in the Sham and without-LIPUS-treatment groups were not treated with LIPUS. All animals were sacrificed after the 3-months phase for further assessment.

### Assessment of cartilage repair

#### Magnetic resonance image (MRI)

All MRI experiments were performed on a 1.5 T GE Signa HDxt clinical whole body scanner. MR images of right knee joints were taken for all experimental rabbits under sedation with intra-muscular injection anesthesia with Zoletil 50 (1.0 ml/kg) for the sham, with-LIPUS-treatment and without-LIPUS-treatment groups. Proton density fast spin echo (PDFSE) pulse sequence was used to acquire sagittal view MR images and to assess the articular cartilage. Scanning parameters for sagittal PDFSE pulse sequence were 4000 ms TR (Repetition time), 30 ms TE (Echo time), 2 mm slice thickness, 2 NEX (Number of excitation), 16 ETL (Echo train length), and 8 cm × 8 cm FOV (Field of view). Under this sequence, MR images could provide good contrast between intermediate-signal articular cartilage and high-signal joint fluid, allowing clear visualization of the cartilage defects. Variation of cartilage defect among different phases was investigated to evaluate the effect of LIPUS on articular cartilage repair.

### Gross morphological evaluation

Morphological changes to the femoral condylar surfaces in the rabbits were assessed before the collection of cartilage samples during operation after sacrifice. The grading scale reported by Yoshiaka [19] was used, which divided the conditions of cartilage into grade I: intact surface; grade II: minimal fibrillation; grade III: overt fibrillation; and grade IV: erosion. Two independent blinded observers performed evaluation of the gross morphology.

### Histopathological evaluation

Histopathological evaluation was performed on the sagittal sections of the operated cartilage in the femoral condyle. Knee joint samples were dissected, fixed in 10% formalin for 24 hr, decalcified by Gooding and Stewart’s fluid (equal volume of 10% formalin and 10% formic acid solution), and embedded in paraffin. H&E stain and Alcian blue stain were employed to observe the injuried cartilage layer and to evaluate the effect of cartilage repair.

The modified Mankin scoring system [20,21] was also used to evaluate cartilage repair histologically. The severity of the cartilage injury lesions was graded on a scale of 0–13, with a combined score of structure (0 – 6 points), matrix staining (0 – 4 points), and cellular abnormalities (0 – 3 points). In this scoring system lower score indicates more healthy cartilage. The histopathological evaluation was performed by two independent blinded observers.

### Statistical analysis of data

The data were presented with mean ± SD. The Fisher-exact Test was used to analyze the results of gross morphology. Histological Mankin scores were analyzed using Wilcoxin signed rank test using SPSS (Statistical Package for Social Science; version 10.0; SPSS, Chicago, IL, USA). Statistical significance was set at p < 0.05.

## Results

**Figure 2:**
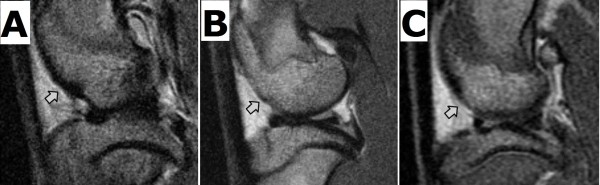
**Sagittal view proton density fast spin echo sequence MR images (TR/TE 4000/30) of operated knee in the with-LIPUS-treatment group.** Subchondral bone demonstrated lower signal intensity, while articular cartilage showed intermediate signal intensity and joint fluid showed high signal intensity. **(A)** Pre-surgery: Intact articular surface of femoral condyle (arrow), and a boundary between joint fluid and articular cartilage were seen. **(B)** Post-surgery: Cartilage defect showed loss of continued articular surface over femoral condyle (arrow). **(C)** Post-LIPUS treatment: Proliferative tissue filled the cartilage defect of femoral condyle (arrow). A boundary between joint fluid and proliferative tissue was seen, which presented intermediate signal intensity.

Three phases (pre-surgery, post-surgery and post-LIPUS-treatment) of sagittal view MR images of experimental rabbit knees were acquired from PDFSE pulse sequence. Under this sequence, the signal intensity of subchondral bone demonstrated lower signal (dark), while that of articular cartilage showed intermediate signal and joint fluid showed high signal intensity (bright). Comparing the MR images from the different phases of rabbits in the with-LIPUS-treatment group, there was a boundary between joint fluid and articular cartilage on the pre-surgery and post-LIPUS-treatment knee, while there was not seen on the post-surgery knee (Figure 2). The intact articular surface of femoral condyle was shown on MR image before surgery (Figure 2A). The cartilage defect over femoral condyle was found on MR image right after surgery (Figure 2B). Some tissue filling cartilage defect of femoral condyle was observed on MR image after LIPUS treatment (Figure 2C). The tissue observed on the cartilage defects in the with-LIPUS-treatment group demonstrated intermediate signal, which similar to articular cartilage.

**Figure 3:**
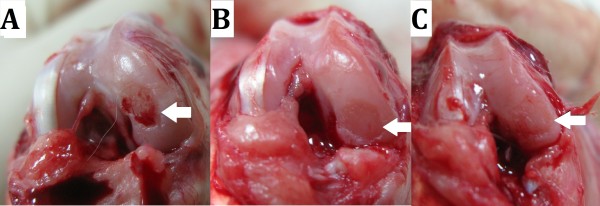
**Gross photography of cartilage defect (white arrow). (A)** Post-surgery: A femoral condylar cartilage defect was created surgically deep to calcification zone until subchondral bone exposure. **(B)** Post-surgery 3-months without LIPUS treatment: persisted exist cartilage defect without obvious coverage of proliferative tissue. **(C)** Post-surgery 3-months with LIPUS treatment: proliferative tissue filled the defect to form a rough surface instead of hollow defect.

**Figure 4:**
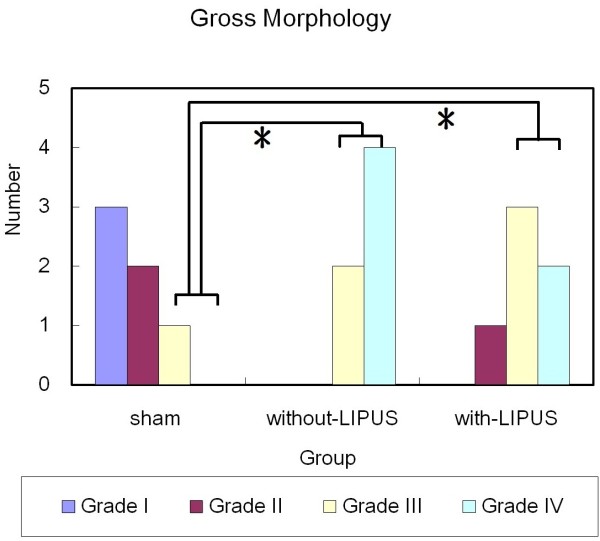
**Gross morphology evaluated by grading scale.** *P < 0.05.

In the evaluation of gross morphology, the surface irregularity became smoother in the with-LIPUS-treatment group than in the without-LIPUS-treatment grossly. Compared with cartilage defect of post-surgery knees, the cartilage defects of the with-LIPUS-treatment group were covered by proliferative tissue to form a rough surface instead of hollow defects. The defects in the without-LIPUS-treatment group still existed and were not covered by proliferative tissue (Figure 3). When considering the severe grades (grades III and IV) of morphological grading scale altogether, statistically significant differences were investigated between the sham group and both the without-LIPUS-treatment group (p < 0.01) and the with-LIPUS-treatment group (p = 0.04) (Figure 4). Both the with-LIPUS-treatment and the without-LIPUS-treatment groups were in worse condition than the sham group. The number of the most severe grade (grade IV) was fewer in with-LIPUS-treatment group than the without-LIPUS-treatment group, but there was no significant difference statistically (p = 0.393). No any grade I (intact cartilage) was observed in the operated groups with-LIPUS-treatment or without-LIPUS-treatment. The best improvement of injuried knees was grade II, which were only observed in the with-LIPUS-treatment group. All experimental knees were revealed severe grades (grade III and IV) in the without-LIPUS-treatment group. However no any significantly statistical differences were found between the operated groups with LIPUS and without LIPUS treatment (p = 0.296).

**Figure 5:**
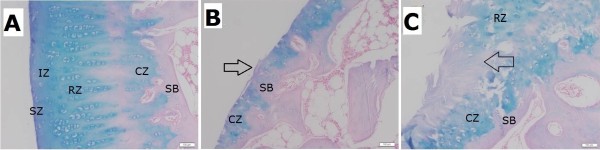
**Typical histological section after 3-months period.** (Alcian blue stain x 100). **(A)** The Sham group: Intact osteochondral architecture was shown. The surface of cartilage layer is smooth and the arrangement of chondrocyte is well. **(B)** The without-LIPUS-treatment group: The cartilage layer was thin because of lack of superficial zone, intermediate zone and radial zone of cartilage layer. Irregular surface without proliferative layer was shown (arrow). **(C)** The with-LIPUS-treatment group: The layers of superficial zone, intermediate zone and radial zone were lost over the cartilage defect, instead of deposition of acellular tissue layer (arrow). SZ: superficial zone; IZ: intermediate zone; RZ: radial zone; CZ: calcification zone; SB: subchondral bone.

**Figure 6:**
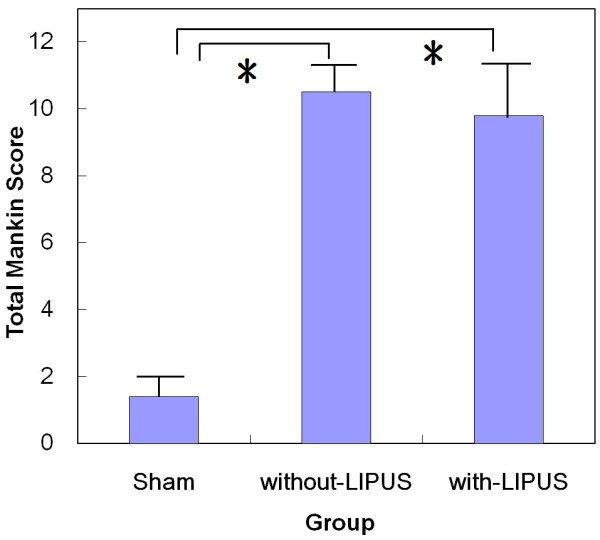
**Total Mankin score for Histopathological evaluation.** *P < 0.05.

In histopathological evaluation, the articular surface was smooth and osteochondral layer was intact arrangement in the sham group (Figure 5A). The articular defect in the without-LIPUS-treatment group showed reduced thickness of cartilage layer and lack of superficial zone, intermediate zone and radial zone (Figure 5B). Not any reparative tissue was observed over the defect. Specifically, in the with-LIPUS-treatment group, the deposited tissue over the cartilage defects was acellularity without up-take under Alcian blue stains in general (Figure 5C) although the surface irregularity became smoother grossly. No new deposition of collagen type II, healthy extra-cellular matrix or proliferation of chondrocytes was observed on the cartilage defect. These histopathological changes were evaluated using the Mankin score qualitatively. The total Mankin score was significantly increased in the groups of with-LIPUS-treatment and without-LIPUS-treatment compared with the sham group (p < 0.01) (Figure 6). The score reduced in the with-LIPUS-treatment group compared with the without-LIPUS-treatment group, but the difference was not statistically significant (p = 0.82).

## Discussion

Many animal models have been developed to investigate the pathogenesis of osteoarthritis, such as spontaneous models in aging animals, enzymatically or chemically induced models, and surgical induced models, as well as ACL transection combined partial meniscus resection [14-17]. In above models, cartilage degenerations as osteoarthritis were induced gradually. Most damage of articular cartilage was relatively mild, similar to early or middle stages of osteoarthritis [12]. Besides, they were difficult to control in the consistency of the cartilage injuries among the animals in the models. In our study, we designed an experimental rabbit model with a uniform articular cartilage defect created surgically to establish the consistency in chondral injuries. Even the location and degree of chondral damage were not characterized histologically. The repair tissues on the defect were evaluated qualitatively. In gross observation of joints after sacrifice, persisted defect over the articular surface without proliferation of healthy tissue was observed in the without-LIPUS-treatment group, whereas the proliferative tissue was observed within the margin of defect in the with-LIPUS-treatment group. Significantly increased number of severe morphological grades in the operated knees without LIPUS treatment indicated the articular defects were severe chondral injuries. Besides Mankin score changes between the sham group and the operated groups also confirmed the severity of the chondral injuries that were created surgically. They indicated that a severe chondral injury has been demonstrated to be feasible to build in this study.

Low-intensity pulsed ultrasound (LIPUS) is a pressure or sound wave with the capability to transfer mechanical energy into biological tissues [21]. It stimulates osteogenesis and fracture healing [3-8]. It produces micromechanical stresses in tissues and elicits an increase in nitric oxide production and activation of hypoxia-inducible factor-1α, thereby inducing the expression of vascular endothelial growth factor levels in osteoblasts [3,22]. The stimulation mechanism of low-intensity ultrasound was suggested to be derived from electrical potentials (piezoelectricity) and not thermal effects. This may lead to the stimulation of angiogenesis, which plays a role in early bone repair and endochondral ossification. Chondrocyte cultures exposed to ultrasound were studied in vitro and showed increased aggrecan mRNA levels and proteoglycan synthesis, suggesting direct ultrasound stimulation of aggrecan expression [11,23]. An increased rate of endochondral ossification was demonstrated in mouse metatarsal rudiments using low-intensity ultrasound [5]. Hence, the LIPUS up-regulating the expression of extracellular matrix proteins and collagens in chondrocytes was characterized. Moreover, it was suggested by Ebisawa that LIPUS has a potential to enhance chondrogenic differentiation, but not cell proliferation [24].

Although many in vitro studies reported the effectiveness of LIPUS on articular cartilage metabolism, only few in vivo studies have been presented. Li X et al. [12] found that LIPUS promoted cartilage repair through the down-regulation of MMP-13 in rabbit knee osteoarthritis with ACL transection model. Naito K et al. [18] also presented that LIPUS increased type II collagen synthesis via induction of type II collagen mRNA expression and activation of chondrocytes in a rat osteoarthritis with ACL and meniscus transection model. In our study, though filling tissue was observed over the chondral defect after LIPUS treatment grossly and under MR images, the layer was acellular tissue without up-take of Alcian blue histologically. No proliferation of chondrocyte or new deposition of normal extra-cellular matrix was observed. The filling tissue may be necrosis or degeneration of remanent debris. No statistically significant difference of the morphology scale and the Mankin score between the with-LIPUS-treatment and the without-LIPUS-treatment groups was assessed. No therapeutic effect of LIPUS on repair of chondral injury was observed in our study, which was not compatible with previous studies. The reason might be the degree of chondral injuries in our model was more severe than previous studies, which presented fibrillation or chondromalacia as mild osteoarthritis induced gradually. The chondral injuries in our model which presented a full-thickness cartilage defect deep to exposure of subchondral bone were too severe injury to repair by stimulation of LIPUS. In other words, the effect of cartilage repair by LIPUS might be less effective in repairing such severe chondral injury extensively.

## Conclusion

We designed a rabbit model of severe chondral injury to investigate the effect of cartilage repair by LIPUS. The results were evaluated using a morphology grading scale, and histopathology. Preliminarily, no significant therapeutic effect of LIPUS was observed in our animal study. On the other hand, the therapeutic effect of LIPUS on human cartilage may be different from rabbit. Besides, the repairing effect may be different in different dosed of LIPUS, which was not test in our study. It needs further study to clarify the effect in the future.

## Competing interests

The authors declare that they have no competing interests.

## Authors’ contributions

SWY and CLK participated in study deign, conducted all experiments and sample analysis. SJC and SMK conceived this study and participated in the design of the experiments, provided assistance for data analysis and statistical analysis and reviewed the manuscript. PCC and IM participated in the development of the study design and data analysis. YTL contributed to the technical work of the model used in the study. All authors read and approved the final manuscript.

## Pre-publication history

The pre-publication history for this paper can be accessed here:

http://www.biomedcentral.com/1471-2474/15/36/prepub
